# Aminopeptidase N1 is involved in *Bacillus thuringiensis* Cry1Ac toxicity in the beet armyworm, *Spodoptera exigua*

**DOI:** 10.1038/srep45007

**Published:** 2017-03-22

**Authors:** Lin Qiu, Songhe Cui, Lang Liu, Boyao Zhang, Weihua Ma, Xiaoping Wang, Chaoliang Lei, Lizhen Chen

**Affiliations:** 1 Hubei Insect Resources Utilization and Sustainable Pest Management Key Laboratory, College of Plant Science and Technology, Huazhong Agricultural University, Wuhan 430070, Hubei, China; 2 College of Life Science, Jilin University, Changchun 130012, Jilin, China

## Abstract

Understanding how insecticidal proteins from the bacterium *Bacillus thuringiensis* (Bt) interact with their hosts is crucial to fully explain the molecular bases of Bt specificity and insecticidal activity. Previous studies support ATP binding cassette transporters (ABCC2/3) and one cadherin-like protein are Cry1Ac functional receptors in the beet armyworm (*Spodoptera exigua*). In this study, a combined one-dimensional gel electrophoresis and immunoblotting approach identified aminopeptidase N (APNs) as putative Cry1Ac binding proteins in the midgut brush border membrane of *S. exigua* larvae. Functional analyses by gene silencing of six different *S. exigua* APN genes (*SeAPN1, SeAPN2, SeAPN3, SeAPN4, SeAPN5* and *SeAPN6*) showed that only suppression of *SeAPN1* resulted in decreased larval susceptibility to Cry1Ac toxin. These results support that *SeAPN1* plays important functional role in Cry1Ac toxicity in *S. exigua*.

The crystal (Cry) proteins from the bacterium *Bacillus thuringiensis* (Bt) are a diverse group of insecticidal proteins employed for the control of numerous pest species from different insect orders^1^. These Cry proteins are active ingredients in Bt sprayable formulations, and *cry* genes have been transformed into transgenic plants for resistance to insect attack. Understanding how Cry toxins interact with their insect hosts is crucial to fully explain the molecular bases of specificity and to develop efficient resistance management tools.

The mode of action of Cry toxins in lepidopteran larvae has been thoroughly investigated^2^. Once the parasporal crystalline bodies containing the Cry proteins are ingested by a susceptible insect, they are solubilized to a protoxin form in the alkaline digestive fluids, and then processed by midgut proteases to an active toxin core. Upon traversing the peritrophic matrix, the activated toxin core binds to specific binding sites on the brush border membrane of the midgut. Binding results in oligomerization and formation of toxin pores that lead to osmotic cell death, compromising the midgut epithelial barrier and allowing resident bacteria to invade the hemocoel to cause septicemia and death of the insect. A number of proteins have been proposed as receptors for the Cry1A family of proteins, including aminopeptidase N (APN), cadherin, ABC transporters and alkaline phosphatase^3^,^4^,^5^,^6^,^7^.

The beet armyworm, *Spodoptera exigua*, has recently become a major economic cotton pest in China^8^,^9^,^10^,^11^, probably due to the reduced pesticide usage attributed to adoption of Bt cotton producing the Cry1Ac protein. Previous studies reported that knockdown of ABCC transporter 2/3 and one cadherin-like protein in *S. exigua* larvae decreased their susceptibility to Cry1Ac^12^,^13^. In the current work, we used a combined one-dimensional (1D) gel electrophoresis and immunoblotting approach to identify APNs as Cry1Ac binding proteins in the midgut of *S. exigua* larvae. Using functional assays by RNA interference (RNAi) to individually silence expression of known *S. exigua* APN genes, we document the identification of APN protein relevant to Cry1Ac intoxication in that insect pest.

## Results

### Binding proteins of Cry1Ac of *S. exigua* BBMV

**Figure 1:**
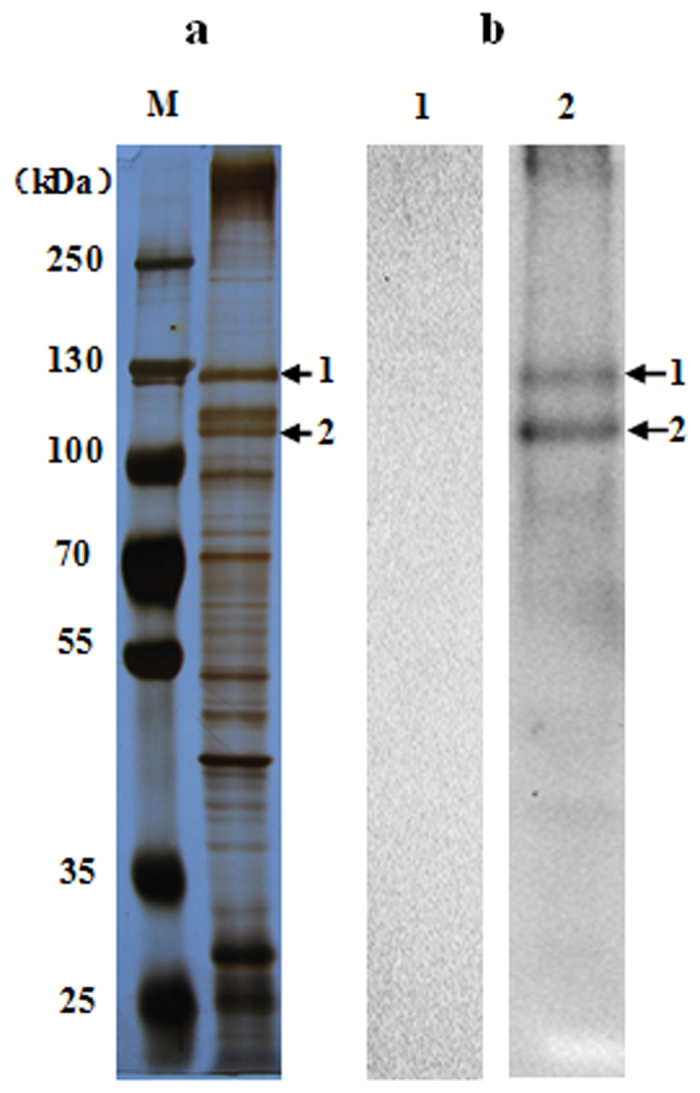
SDS-PAGE analysis of BBMV solubilized protein from *S. exigua* and ligand blotting with Cry1Ac. (**a**) Total protein silver staining detection of separated *S. exigua* BBMV proteins. (**b**) *S. exigua* BBMV proteins binding Cry1Ac in ligand blots, as detected with Cry1Ac antisera. Panel 1, blotting assay without Cry1Ac, Panel 2, blotting assay with Cry1Ac. Arrows indicate detected Cry1Ac-binding protein bands.

Ligand blots of midgut brush border membrane proteins from *S. exigua* larvae. Figure 1a identified two prominent Cry1Ac-binding protein bands of about 110- and 130-kDa in size, respectively, which were numbered as bands 1 and 2 (Fig. 1b, panel 2). The specificity of the anti-Cry1Ac antisera used for ligand blotting was confirmed by the lack of cross-reactivity in blots with no Cry1Ac (Fig. 1b, panel 1). The Cry1Ac binding bands were excised and submitted to LC-MS/MS analysis and protein database searching. Parameters used for protein identification included at least two unique peptides detected and molecular weight similar to the Cry1Ac protein bands. The list of detected proteins fulfilling these conditions in each Cry1Ac-binding band is presented in Supplementary Table S1. Among all these proteins, the most abundant in both bands 1 and 2 were N-aminopeptidases (APNs) from *S. exigua* (Table 1). Consequently, we focused our analyses on testing the functional Cry1Ac-receptor role of *S. exigua* APNs (SeAPNs).

### Functional Cry1Ac receptor assays of APNs in *S. exigua*

**Figure 2:**
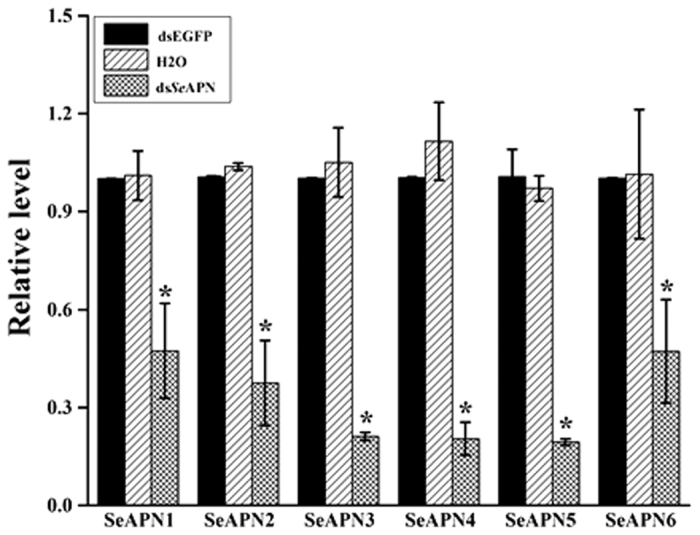
RNA interference knockdown of *Se*APN transcripts in *S. exigua* larvae. Relative levels of *Se*APN transcripts were determined by qRT-PCR of *S. exigua* larvae fed artificial diet overlaid with either water or dsEGFP as controls, or dsRNA targeting each specific *Se*APN. The *Se*GAPDH and *Se*RpL10 housekeeping genes were used to normalize transcript levels. Asterisks indicate significant differences (ANOVA followed by Tukey’s HSD posthoc test, P < 0.05).

**Figure 3:**
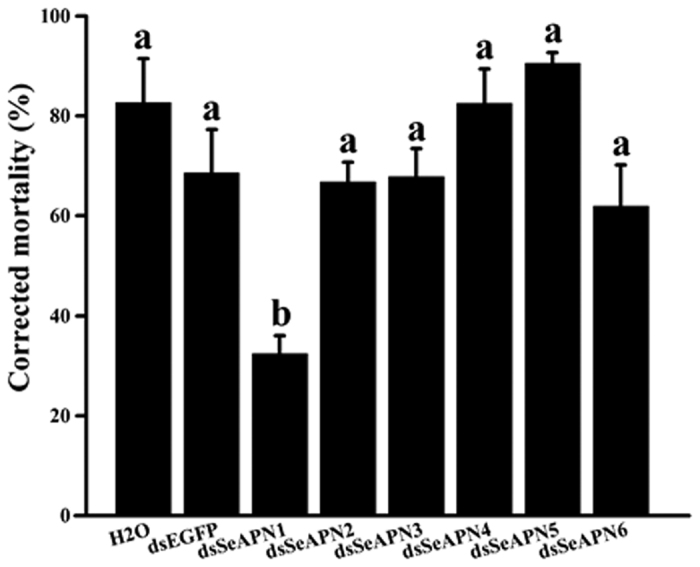
Corrected mortality by Cry1Ac in *S. exigua* larvae treated with dsRNA. Larvae were fed on diet overlaid with dsRNA targeting EGFP, *SeAPN1, SeAPN2, SeAPN3, SeAPN4, SeAPN5 or SeAPN6*, and then they were exposed to diet contaminated with 3 μg/cm^2^ of Cry1Ac. Bars denotes standard error of the mean calculated from five replicates. Different letters on top of bars indicate significant differences (ANOVA followed by Tukey’s HSD posthoc test, P < 0.05).

Phylogenetic analyses identified six *SeAPN* genes (*SeAPN1* to *SeAPN6*) belonging to the same number of APN families^14^, which were selected for further testing. To test their putative Cry1Ac receptor role, we used a gene silencing approach by RNA interference (RNAi) through ingestion of double-stranded RNA (dsRNA) targeting each *SeAPN* gene. After ingestion of purified dsRNAs specific to *SeAPN1, SeAPN2, SeAPN3, SeAPN4, SeAPN5* or *SeAPN6* for 48 h, the transcript levels for these genes were significantly reduced by 53%, 62%, 79%, 80.6%, 81% and 53%, respectively, when compared to larvae fed on dsEGFP or water as controls (Fig. 2). Subsequent feeding larvae exposed to dsRNA to a diet overlaid with 3 μg/cm^2^ of Cry1Ac resulted in 83% and 68% mortality in the water and dsEGFP controls, respectively. In contrast, mortality was 32% (dsAPN1), 67% (dsAPN2), 68% (dsAPN3), 82% (dsAPN4), 96% (dsAPN5), and 62% (dsAPN6) in the experimental treatments (Fig. 3). Statistical analyses (ANOVA, P < 0.05) revealed that the only treatment affecting Cry1Ac susceptibility was feeding on dsSeAPN1 when compared to the water or dsEGFP treatments.

## Discussion

Aminopeptidases N (APNs) are a class of metalloenzymes widely present in the apical membranes of the insect midgut that remove neutral amino acids from the N-terminus of polypeptides^4^. A number of studies support APNs as binding proteins for Cry toxins in lepidopteran and dipteran insects^15^,^16^,^17^,^18^,^19^,^20^,^21^. For instance, expression of a *Manduca sexta* APN gene in transgenic *Drosophila* resulted in susceptibility to Cry1Ac toxin^22^. Silencing of APNs expression results in reduced susceptibility to Cry1C in *S. litura*^23^, to Cry1Ac in *H. armigera*^24^, or to Cry4Ba in *A. aegypti*^25^. Moreover, resistance to Cry1Ac was correlated with down-regulation and deletion mutations in APN1 genes in *Trichoplusia ni* and *H. armigera*, respectively^26^,^27^. Very recently, APN1 has been identified as a Cry1Ac receptor in *H. zea*^28^, while APN1 and APN2 genes were reported as Cry11A receptors in *A. aegypti*^20^,^29^. Two partial APN fragments from *Anopheles gambiae* had inhibitory effects on the larval susceptibility to Cry11B toxin^19^.

In *S. exigua*, a total of six APN genes were identified in a previous study^14^. Silencing of *SeAPN1, SeAPN3* or *SeAPN6* expression was shown to reduced susceptibility to Cry1Ca^14^. Moreover, *S. exigua* resistance to Cry1Ca was associated with lack of expression of a *SeAPN1* gene^30^. In the present work, we present the identification of *SeAPN1* as a Cry1Ac receptor, and data supporting that other SeAPNs do not serve as receptors for this toxin. Together with previous reports, these data support that SeAPN1 is a common functional receptor for Cry1Ac and Cry1Ca toxins^30^. This observation would suggest that cross-resistance between Cry1Ac and Cry1Ca in *S. exigua* is likely. In agreement with this hypothesis, cross-resistance was observed between Cry1Ab and Cry1Ca in *S. exigua* larvae after selection with toxin^31^. The possible reason of this cross-resistance might be that the two toxins share same binding site of *SeAPN1*, while further work would be needed to test this hypothesis, sharing of binding proteins between Cry1 and Cry1Ca proteins would have important consequences for resistance management tactics, as these proteins have been proposed as candidates for gene pyramiding in transgenic crops^32^,^33^.

Our data also clearly support that SeAPN1 is the only SeAPN functioning as a Cry1Ac receptor. Other reports also support APN1 proteins as receptors for Cry1Ac in *T. ni*^27^, *M. sexta*^34^ and *H. armigera*^26^. However, there is evidence for alternative APNs interacting with Cry1Ac in other lepidopteran insects. For instance, APN2 binds Cry1Ac in *H. armigera*^35^, but not in *Lymantria dispar*^36^, *M. sexta, P. xylostella* or *B. mori*^16^,^37^,^38^. Both APN3 and APN5 can bind Cry1Ac in *H. armigera*^39^ and *P. xylostella*^40^, but their involvement in Cry1Ac resistance remains to be confirmed. In contrast, there is no evidence supporting a Cry1Ac receptor role for APN4, APN6, APN7 or APN8 proteins. Specific glycosylation or sequence attributes may explain this specificity of Cry1Ac for some APN proteins.

In our combined ligand blotting and MS/MS analyses we identified two protein bands as SeAPN1 and SeAPN3. In addition to SeAPNs, we also detected other proteins in the Cry1Ac-binding bands that may specifically interact with Cry1Ac. For example, *S. exigua* cadherin peptide was detected in band 1, albeit with low probability, and cadherins have been demonstrated to act as Cry1Ac receptors in previous studies^12^,^41^, The functional role for these alternative proteins needs to be examined. Based on relative abundance and the results from gene silencing, the present work identifies SeAPN1 as the only SeAPN acting as a functional Cry1Ac receptor in *S. exigua* larvae. The potential sharing of this receptor needs to be further explored to evaluate risks of resistance evolution for pyramided Cry1A and Cry1Ca genes in transgenic crops.

## Materials and Methods

### Insect rearing, midgut dissection and BBMV preparation

*S. exigua* larvae were collected at the campus greenhouse of the Huazhong Agricultural University in June 2012 and reared without exposure to Cry toxins. Insects were maintained at 28 ± 1 °C, a 14 L:10D photoperiod, and 70–80% relative humidity. Larvae were reared on an artificial diet^42^ and adults fed on 10% sucrose. Actively feeding fourth-instar larvae were chilled for 5 minutes on ice and dissected, the midgut tissue was cleaned from trachea, Malpighian tubules, peritrophic membrane and food bolus and rinsed briefly in ice-cold MET buffer (300 mM Mannitol, 17 mM Tris-HCl, 5 mM EGTA, pH 7.5). Dissected midguts were stored frozen at −80 °C until used.

BBMV were prepared from the dissected midguts by the differential magnesium precipitation method^43^. Briefly, midguts were homogenized in nine volume of midgut weight MET buffer containing 1 mM Phenylmethanesulfonyl fluoride (PMSF) using a tissue homogenizer, an equal volume of 24 mM MgCl_2_ was added and samples were incubated on ice for 15 min before centrifugation at 2,500 g for 15 min. This step was repeated three times, and the combined supernatant was collected and centrifuged at 30,000 g for 30 min. The final BBMV pellet was resuspended in ice-cold buffer (10 mM HEPES, 130 mM KCl, 10% glycerol, pH 7.5) containing 1 mM PMSF^43^. Protein concentration was determined by the method of Bradford^44^ with bovine serum albumin (BSA) as a standard.

### Ligand blotting and mass spectrometry

Proteins of *S. exigua* BBMV (10 μg) were separated by 8% SDS-PAGE and transferred 25 minutes to PVDF filters at 15 V constant voltage. After blocking in PBST buffer (135 mM NaCl, 2 mM KCl, 10 mM Na_2_HPO_4_, 1.7 mM KH_2_PO_4_, pH 7.5, 0.1% Tween-20) containing 5% (w/v) skim milk for 2 h, filters were incubated with 0.3 μg/ml of activated Cry1Ac for 2 h at room temperature. A control experiment was performed without incubation with Cy1Ac toxin. The filters were washed in PBST buffer three times followed by probing with a 1:3,500 dilution of polyclonal antibody to Cry1Ac for 2 h. After washing as above, the membranes were incubated in 1:5,000 diluted goat anti-rabbit IgG horseradish peroxidase (HRP)-linked antibody. The filters were developed with an ECL kit (Fermentas/Thermo Fisher Scientific, Waltham, MA USA) following manufacturer’s recommendations.

After ligand blotting, the gel bands observed to bind Cry1Ac were excised and rinsed in destaining solution (30% acetonitrile/100 mM NH_4_HCO_3_). The gel bands were then incubated with 100 mM Dithiothreitol (DTT) at 56 °C for 30 minutes, treated with 200 mM indole-3-acetic acid (IAA) after abandon supernatant, and then incubated with 100 mM NH_4_HCO_3_ then remove liquid. As a last step, samples were treated with 100% acetonitrile for 5 minutes and freeze dried before digestion with 2.5–10 ng/μg trypsin for 24 hours at 37 °C and analysis by liquid chromatography-electrospray ionization tandem mass spectrometry (LC-ESI-MS/MS) at the Shanghai Life Science Research Institute (China Academy of Sciences, Shanghai, China). The mass spectrometry results were queried to the uniprot database using the Mascot2.2 software.

### RNA interference of *SeAPNs*

The pET-2P plasmid was used to produce double-stranded RNAs targeting *SeAPNs* (dsSeRNA) and *EGFP* (dsEGFP), as described by Ren *et al*.^14^. The primers used in cloning the dsRNA fragments are listed in Table 2. Amplicons were purified and digested with restriction enzymes (Table 2), and then ligated into the previously digested pET-2P, to generate pET-2P/*SeAPN* and pET-2P/*EGFP* dsRNAs plasmids. Correct inserts were confirmed by sequencing at Genscript Biology Company, Nanjing, China. For dsRNA expression, 200 ng of plasmid DNA was transformed into *Escherichia coli* HT115 (DE3) competent cells, positive clones were cultured in 500 ml LB medium and induced to express dsRNA by adding 0.4 mM isopropyl-D-thiogalactoside (IPTG). The same methods described by Timmons *et al*.^45^ and Dong *et al*.^46^ were used to extract dsRNA from aliquots of bacteria, and the size of dsRNA was confirmed by electrophoresis on a 1% agarose gel.

### Bioassay

Newly hatched *S. exigua* larvae were fed artificial diet overlaid with 50 μg/cm^2^ of *dsSeAPNs, dsEGFP* or water for 48 h at 27 °C. Larvae were then transferred to wells of a 6-well plate where they were allowed to feed for 7 days on artificial diet contaminated with 3 μg/cm^2^ of activated Cry1Ac toxin (equivalent to the LC_70_ value according to preliminary experiment), or on diet contaminated with water as a control. A total of 120 larvae were used for five replicated bioassays for each treatment. To monitor the silencing efficiency for each target gene, 15 larvae whole body from each replicate, 3 replicates for each dsRNA treatment were used to extract total RNA. The relative differences of target gene expression level were detected by qRT-PCR with the primers presented in Table 2, which were designed in the NCBI profile server (http://www.ncbi.nlm.nih.gov/tools/primer-blast). The *SeGAPDH* and *SeRpL10*^47^,^48^ genes were used as reference for normalization. The qPCR protocol followed was described elsewhere^41^.

### Data analysis

Abbott’s formula was used to calculate the larval corrected mortalities^49^, means and variances of treatments were analyzed by one-way ANOVA using SPSS for Windows (SPSS 18.0, Chicago, IL, USA). Quantitative expression data were analyzed by the 2^−∆∆Ct^ method^50^.

## Additional Information

**How to cite this article:** Qiu, L. *et al*. Aminopeptidase N1 is involved in *Bacillus thuringiensis* Cry1Ac toxicity in the beet armyworm, *Spodoptera exigua. Sci. Rep.* **7**, 45007; doi: 10.1038/srep45007 (2017).

**Publisher's note:** Springer Nature remains neutral with regard to jurisdictional claims in published maps and institutional affiliations.

## Supplementary Material

Supplementary Information

## Figures and Tables

**Table 1:** Most abundant Cry1Ac binding proteins identified in BBMV from *Spodoptera exigua*.

Band^a^	Accession number^b^	PepCount	Unique PepCount	MW	Top ranking match	Species
1	Q4G6A5	35	26	114.9	Midgut class 1 aminopeptidase N	*Spodoptera exigua*
2	Q5UVJ2	28	25	113.9	Aminopeptidase N	*Spodoptera exigua*

^a^Band number corresponding to Fig. 1b, panel 2.

^b^Uniprot database accession number.

**Table 2:** Nucleotide primers used to amplify cDNA fragments for dsRNA synthesis, and for quantitative real-time PCR analysis of RNAi knockdown.

Primer	Primer Sequence (5′-3′)
**Primers for dsRNA Synthesizing**	
*Se*APN1 Fw	actGAATTCCCCTCAACGACCATTCACTATC^1^
*Se*APN1 Rv	actGAATTCGAAGGAGTCGGATAGCAAGGA^1^
*Se*APN2 Fw	actGAATTCTATTGGCAGTGGTGAAGAGC^1^
*Se*APN2 Rv	actGAATTCCATAACAACAGTCTTACAGGAACC^1^
*Se*APN3 Fw	actGCGGCCGCCCGAATGACAGAACATCTCCTT^2^
*Se*APN3 Rv	atcACTAGTTATCACCCACCGAGATGGAC^3^
*Se*APN4 Fw	actGAATTCTTCACAAATCGGCTTAGGAGG^1^
*Se*APN4 Rv	actGAATTCCGAGACGAAAACAACATTAAATTG^1^
*Se*APN5 Fw	actGAATTCTGGCTACTTGGATGAGGAAGG^1^
*Se*APN5 Rv	actGAATTCGGTAATGAACTGTTCCAGTGATCA^1^
*Se*APN6 Fw	actGAATTCTTCAGGAATCTTGGGACCG^1^
*Se*APN6 Rv	actGAATTCGATAGCGTTCTTTGCTGTTGC^1^
**Performing the qRT-PCR**	
*Se*APN1 qRT-PCR Fw	GGGTGTACTGCGGTGGTCTT
*Se*APN1 qRT-PCR Rv	CAACCTGCTGCACCAAGCAT
*Se*APN2 qRT-PCR Fw	CTGGTGTACTGCGCTGGTCT
*Se*APN2 qRT-PCR Rv	TGCTCGCTGTGATCTTGGCT
*Se*APN3 qRT-PCR Fw	CGCTGCTGTCTCAGGCAATG
*Se*APN3 qRT-PCR Rv	GCTGCATTCCTCAACCTGGC
*Se*APN4 qRT-PCR Fw	AATGCATACGGCATCGGCAC
*Se*APN4 qRT-PCR Rv	ACTGTCGAACACAGCCCCAA
*Se*APN5 qRT-PCR Fw	CTGTGAGGGTCTCCGAGCTG
*Se*APN5 qRT-PCR Rv	TGCATCCCAGGGCTCTCAAC
*Se*APN6 qRT-PCR Fw	ACGGTCTTGCTGACCACGTT
*Se*APN6 qRT-PCR Rv	AGTTCCGGCAGAAGCCCAAT
*Se*GAPDH qRT-PCR Fw	CTGAGGAACAGGTCGTGTCA
*Se*GAPDH qRT-PCR Rv	TTCAGAGAGATACCGGCAGCA
*Se*RpL10 qRT-PCR Fw	CTCTGCGTCGTGCCAAGTTC
*Se*RpL10 qRT-PCR Rv	CCTCACGCAGCTTCTCGAAT

^1^Underlined sequence indicates position of the *EcoR* I endonuclease site.

^2^Underlined sequence indicates position of the *Not* I endonuclease site.

^3^Underlined sequence indicates position of the *Spe* I endonuclease site.
