# Supplementary material for: Aminopeptidase N1 is involved in Bacillus thuringiensis Cry1Ac toxicity in the beet armyworm, Spodoptera exigua
Source: Sci Rep. 2017 Mar 22;7:45007. doi: 10.1038/srep45007 (PMC5361178; doi:10.1038/srep45007)
Supplement: Supplementary Information [file srep45007-s1.pdf]

SUPPLEMENTARY INFORMATION

Aminopeptidase N1 is involved in *Bacillus thuringiensis* Cry1Ac toxicity in the beet armyworm, *Spodoptera exigua*

Lin Qiu[1, #], Songhe Cui[2, #], Lang Liu[1], Boyao Zhang[1], Weihua Ma[1], Xiaoping Wang[1], Chaoliang Lei[1], Lizhen Chen[1, *]

[1]Hubei Insect Resources Utilization and Sustainable Pest Management Key Laboratory, College of Plant Science and Technology, Huazhong Agricultural University, Wuhan, 430070, Hubei, China;

[2]College of Life Science, Jilin University, Changchun, 130012, Jilin, China.

[#]These authors contribute equally in this work.

*Corresponding author: Lizhen Chen

Email: lzchen@mail.hzau.edu.cn

Tel: +86-27-87287207

Fax: +86-27-87287207

Supplementary material

Determining the specificity of polyclonal Antibody

Ten micrograms pure activated Cry1Ac toxin (EnviroLogix Inc., Portland, ME, USA) were added into lane of the 8% SDS-PAGE, the Cry protein was separated and transferred to polyvinylidene difluoride (PVDF) membrane using a Trans-Blot SD Semi-Dry Transfer Cell (Bio-Rad, Hercules, CA, USA). Blocking with PBST buffer (135 mM NaCl, 2 mM KCl, 10 mM Na_2HPO_4, 1.7 mM KH_2PO_4, 0.1% Tween-20, pH 7.5) containing 5% (w/v) skim milk for 2 h, membrane was incubated with Cry1Ac polyclonal antibody (1:3,500). After three times washing (10 min each time), membrane was then incubated with goat anti-rabbit IgG horseradish peroxidase (HRP)-linked antibody (Genscript Biology Company). After final washes, membrane was developed using ECL chemiluminescence detection kit (Fermentas/Thermo Fisher Scientific, Waltham, MA USA) following manufacturer's recommendations.

Supplementary Figure

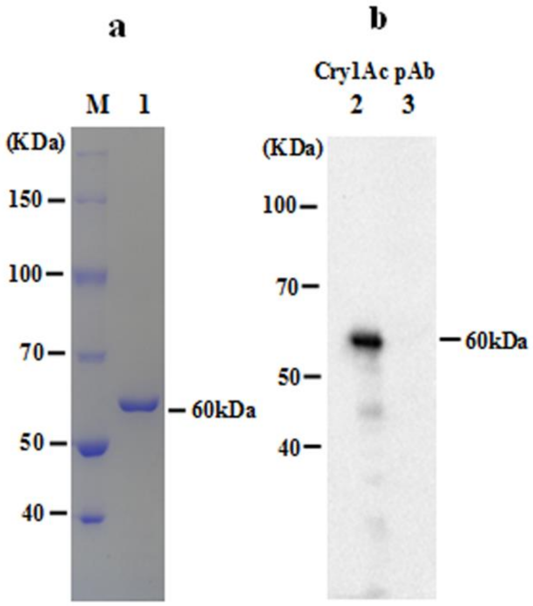

Figure S1. Electrophoretic and western blot analysis of Cry1Ac toxin used for ligand blotting. Purified Cry1Ac toxin (10 μg) was separated by 8% SDS-PAGE, and gels were stained (a) or transferred to PVDF filters for Western blotting with Cry1Ac antibody (b). Lanes 1, 2 = Cry1Ac, lane 3= Cry2Aa.

Supplementary Table S1: Summary of the identifications of cry1Ac binding proteins in the BBMV of the Beet armyworm using uniprot database and Mascot2.2 software. a: Band numbers are related to Figure 1. b: Proteins in the uniprot database for which significant peptide mass matching or sequence similarity was observed.

Band number[a]	Accession number[b]	PepCount Sequences	Unique PepCount	MW	Top ranking match	Species
1	Q4G6A5	35	26	114.9	Midgut class 1 aminopeptidase N	*Spodoptera exigua*
	C4P7G5	13	13	96.1	Myosin heavy chain (Fragment)	*Bombyx mandarina*
	H9JFZ5	13	11	132.4	Pyruvate carboxylase	*Bombyx mori*
	Q9U5M9	9	9	95.9	Vacuolar ATPase subunit a	*Manduca sexta*
	H6WC44	6	6	126.5	Plasma membrane calcium ATPase	*Spodoptera littoralis*
	G6DDY8	6	5	38.6	Phosphate transport protein	*Danaus plexippus*
	I4DMQ1	5	4	38.8	Mitochondrial phosphate carrier protein	*Papilio polytes*
	A2ICN6	4	4	32.9	ADP/ATP translocase	*Dendrolimus punctatus*
	S4PH37	4	4	275.8	Alpha Spectrin	*Pararge aegeria*
	H9JIQ1	4	3	180.6	Protein mesh	*Bombyx mori*
	E9LP50	3	3	142.8	ATP-binding cassette sub-family B member 1	*Trichoplusia ni*
	J7EMA0	3	3	196.4	Cadherin	*Spodoptera exigua*
	S4NXF8	3	3	103.4	Hypoxia up-regulated protein 1	*Pararge aegeria*
2	Q5UVJ2	28	25	113.9	Aminopeptidase N	*Spodoptera exigua*
	H9JCE9	7	6	105.9	Lon protease homolog, mitochondrial	*Bombyx mori*
	Q9U5M9	6	6	95.9	Vacuolar ATPase subunit A	*Manduca sexta*
	H9JFZ5	5	5	132.3	Pyruvate carboxylase	*Bombyx mori*
	D9N4J4	4	4	98.9	Tudor staphylococcus/micrococcal nuclease	*Bombyx mori*
	G6DKT2	4	4	26.4	Myosin-IB	*Danaus plexippus*
	I4DMQ1	4	3	38.8	Mitochondrial phosphate carrier protein	*Papilio polytes*
	G6DDY8	3	3	38.6	Phosphate transport protein	*Danaus plexippus*
	G6DIQ9	3	3	100.3	Tudor micrococcal nuclease	*Danaus plexippus*
	S4P6J9	3	3	134.4	Vigilin (Fragment)	*Pararge aegeria*
	S4PY67	3	3	99.8	Tudor-SN	*Pararge aegeria*

Supplementary Table S2: Specifications for optimized qRT-PCR amplification of *Spodoptera exigua APN1, APN2, APN3, APN4, APN5, APN6, GAPDH* and *RpL10*.

Gene name	Efficiency	R2	Slope
SeRpL10	101.1%	0.996	-3.296
SeGAPDH	100.3%	0.999	-3.316
Se APN1	97.4%	0.986	-3.386
Se APN2	100.3%	0.989	-3.315
Se APN3	100.8%	0.994	-3.303
Se APN4	95.3%	0.998	-3.441
Se APN5	93.5%	0.995	-3.490
Se APN6	96.3%	0.981	-3.415
